# Depressive symptoms and perception of risk during the first wave of the COVID‐19 pandemic: A web‐based cross‐country comparative survey

**DOI:** 10.1111/1467-9566.13350

**Published:** 2021-07-26

**Authors:** Marco Terraneo, Linda Lombi, Hannah Bradby

**Affiliations:** ^1^ Department of Sociology and Social Research Università of Milano‐Bicocca Milan Italy; ^2^ Department of Sociology Università Cattolica del Sacro Cuore Milan Italy; ^3^ Department of Sociology Uppsala University Uppsala Sweden

**Keywords:** COVID‐19, mental health, pandemic, public health, risk perception

## Abstract

Evidence is accumulating of the negative impact of the COVID‐19 pandemic and related public health measures on mental health. In this emergent field, there has been little research into the role of risk perception on depressive symptoms and the contribution of health‐care resources to model risk perception and mental health. The aim of this paper is to describe the relationship between individual‐level perception of risk and depression, controlling for a set of confounders and for country‐level heterogeneity. A cross‐sectional and observational online survey was conducted using a non‐probability snowball sampling technique. We use data on 11,340 respondents, living in six European countries (Italy, Sweden, United Kingdom, France, Poland, Czech Republic) who completed survey questionnaires during the first months of the pandemic. We used a fixed‐effect approach, which included individual and macro‐level variables. The findings suggest that a high proportion of people suffering from depression and heightened risk perception is positively associated with reporting depressive symptoms, even if this relationship varies significantly between countries. Moreover, the association is moderated by contextual factors including health‐care expenditure as a percentage of Gross Domestic Product, hospital beds for acute care, and number of medical specialists per head of population. Investment in health care offers a concrete means of protecting the mental health of a population living under pandemic restrictions.

**Abbreviations**
CI: confidence interval
COVID‐19: SARS‐CoV‐2
DSM‐IV: Diagnostic and Statistical Manual of Mental Disorders (Version IV)
GDP: Gross Domestic Product
LR test: Likelihood‐ratio test
M1: Fixed effects estimation of Model 1
M2: Fixed effects estimation of Model 2
M3: Fixed effects estimation of Model 3
M4_BED: Fixed effects estimation of Model 4 (includes the interaction between risk perception and acute hospital beds
M4_EXP: Fixed effects estimation of Model 4 (interaction between risk perception and the healthcare expenditure)
M4_MED: Fixed effects estimation of Model 4 (includes the interaction between risk perception and number of medical specialists)
OECD: Organization for Economic Co‐operation and Development
PHQ‐8: Personal Health Questionnaire Depression Scale
R^2^
: Coefficient of determination
US: United States of America
UK: United Kingdom
WHO: World Health Organization

## INTRODUCTION

The COVID‐19 pandemic is the most severe global health challenge of the new millennium. From early in 2020 governments introduced measures to reduce the number of infections, including physical distancing (misnamed ‘social distancing’), limits to freedom of movement intra‐ and internationally, and the closure of social spaces. The efficacy of such interventions continues to be debated, while the challenges that such measures bring, collectively and individually, are also of interest to researchers and policymakers. While measuring the effects of the public health precautions against infection and mortality has been central to society's response to COVID‐19, far less attention has been given to measuring other costs and benefits associated with the widespread adoption of these precautions. This paper investigates the costs in terms of mental health.

Early single‐country studies have shown the negative psychological impact of COVID‐19 on the general population (Wang et al., 2020; Rajkumar, 2020), confirming the severe health impacts documented in previous pandemics (Brooks et al., 2020). The high prevalence of symptoms of psychological distress and disorder associated with the COVID‐19 pandemic has been documented with the worsening of mental health conditions attributed to two related factors. First, the fear of illness (fear of falling ill and of death for oneself and others) and of its possible consequences (social exclusion due to quarantine, loss of income) (Brooks et al., 2020). Second, the development of psychological problems related to loneliness, isolation, loss of employment, social and physical distancing (Fiorillo & Gorwood, 2020).

Mental health during a pandemic (as at other times) depends on individual (age, gender, educational qualification, working conditions, family status), as well as structural factors (for example, the measures taken in a specific country to cope with the pandemic) and a mixture of the two (for instance, how employment is affected by public health measures).

In China, Huang and Zhao (2020) conducted a web‐based survey collecting data from 7236 Chinese citizens to assess psychological symptoms during the lockdown in China in February 2020. Their findings suggest that the overall prevalence of anxiety disorders, depressive symptoms, and sleep quality were, respectively, 35.1%, 20.1%, and 18.2%. In Europe, of the 1005 individuals that Pieh et al. (2020) interviewed in Austria during the lockdown, 21% presented moderate depressive symptoms, measured by the Patient Health Questionnaire (PHQ‐9). Their findings suggest that the COVID‐19 pandemic and lockdown have been particularly stressful for younger adults (<35 years), women, people without work, and on a low income. A web survey of the Italian general population (18,147 respondents) reported a strong association between psychological disorders and COVID‐19‐related risk factors, with a high rate of Post‐Traumatic Stress Syndrome (37.1%), severe depressive symptoms (17.3%), severe anxiety symptoms (20.8%), and severe insomnia symptoms (7.3%) (Rossi et al., 2020).

A nationally representative sample of 10,368 U.S. adults surveyed online during March 2020, showed that more than 25% of the sample reported moderate to severe anxiety symptoms (Fitzpatrick et al., 2020).

The forced isolation of lock‐down and quarantine can amplify psychological distress. Many studies reported negative psychological effects due to quarantine, including post‐traumatic stress symptoms, fear, confusion, and anger (Brooks et al., 2020), with stigma a frequent predictor of psychological distress (Mackoli & Mackolil, 2020).

To summarize, recent research has provided preliminary survey evidence that significantly elevated symptomatology, including raised levels of depression, anxiety, general stress, and post‐traumatic stress, is related to COVID‐19. The available evidence suggests that mental distress is associated with being a woman, younger than 35 years, a singleton, a parent living with children, unemployed, suffering from prior mental disorders, and a health‐care worker highly exposed to the virus. Several studies have reported a strong association between psychological distress, anxiety, depression, and exposure to news media.

Psychological research has investigated virus‐related fear (Alyami et al., 2020; Bakioglu et al., 2020; Doshi et al., 2020; Fitzpatrick et al., 2020) as a dimension of distress, but there has been little discussion of the role of risk perception on mental health during the pandemic. Moreover, the contribution of health‐care resources to model the relationship between risk perception and depressive symptoms has scarcely been investigated.

### Theories of risk in a sociological perspective

Risk is a pervasive feature of modernity, taken as a distinctive feature and for some, the ‘dark side’ of contemporaneity (Luhmann, 1991). Late modernity has been associated with the experience of generalized anxiety, manifest through a sense of impending disaster and a normalized expectation of catastrophe (Lash, 2000). Risk in contemporary society is a sense of threat, not only to individuals, but also to the stability of groups, with the perception of risk and the practices to control that risk, being realized through a cultural process of elaboration (Douglas, 1992). Risks are always both ‘constructed’ and ‘real’, in the sense that they are ‘comprised of complex networks of materialities, procedures, regulations, discourses and strategies – and emotions’ and involve ‘an incipient rather than a realized threat or danger’ which ‘is about projecting ideas into the future, about imagining the consequences of an action or event’ (Lupton, 2013, p. 638).

On the emotional level, the negative emotion associated with risk is fear (Harper et al., 2020). Fear can be interpreted as an adaptive response in the presence of danger, a rational reaction to a life‐threatening event and a prompt for self‐preservation (Giddens, 1990). Fear of illness has been widely documented, especially the fear of contagion from infectious disease (Brown & Lees‐Haley, 1992). With specific regard to COVID‐19, several studies have shown how higher levels of perceived risk are associated with higher levels of fear of contagion and worse mental health (Sloan et al., 2020; Yıldırım et al., 2020).

Fears around risk (Harper et al., 2020) are enhanced by the uncertainty of global catastrophe and risks that involve future generations (WHO, 2002). People's mental health tends to get worse over the period of a crisis (Ding et al., 2020) and the negative impact is associated with a perception of increased risk, due to worsening living conditions, the uncertainty of the future, and widespread fear. Risk perceptions are based on processing a diverse array of information relating to risk factors, informed by experience, beliefs, and meaning systems within a specific cultural context (WHO, 2002). Contextual factors such as societal values, support, health‐care arrangements, etc. can mitigate or enhance the effect of risk perception at the individual level, with a consequent effect on wellbeing. Specifically, considering the central role of health‐care services in responding to the pandemic in terms of curative care, as well as information about infection control, in this work we pay attention to macro‐level organizational and institutional aspects of the health system as factors that intervene with respect to individual wellbeing.

The conceptual framework informing this investigation of the first period of pandemic can be summarized as follows: the COVID‐19 pandemic is considered to be a health, social, and economic global crisis, which significantly increases people's risk perception about their own and others’ health and wellbeing. In turn, higher perception of risk is associated with poorer mental health, as indicated by more depressive symptoms, and this relationship could be modified (positively or negatively) by health‐care system performance.

The contribution of this study is threefold. First, it provides harmonized data, for six European countries, gathered during the first wave of the pandemic, with different experience of the impact of the pandemic in terms of contagion and death rates, and different strategies of response, all of which are factors which could influence the relationship between risk perception and depression. Second, it explores how risk perception has affected the mental health of people during the first wave of COVID‐19, including both individual and contextual dimensions. Third, it investigates the impact of health‐care resources to model risk perception and depressive symptoms.

## DATA AND METHOD

### Study design and participants

A cross‐sectional and observational online survey was conducted using a non‐probability snowball sampling technique. The online questionnaire was developed using the software Qualtrics©. The presentation of the study and the invitation to participate were disseminated through Social Media, asking users to share the link with their contacts.

A total of 11,340 respondents, living in six European countries (Italy, Sweden, United Kingdom, France, Poland, and Czech Republic) filled out the survey, but participants with more than 20% missing data were excluded from analysis. The final sample thus consists of 9541 cases. Data were collected between 27th March and 10th July 2020.

As we have a non‐probability sample, we apply a post‐stratification vector to match the known population distribution according to auxiliary variables. The data samples were re‐weighted by country to reflect the gender, age, and educational structures of the general population. Post‐stratification is probably the most popular technique for adjustments for Web‐survey (Schonlau & Couper, 2017). All analyses in this study used weighted data.

The online survey collected demographic data, information on COVID‐19 experience, media sources of COVID‐19 information, COVID‐19 risk perception, lifestyle behaviours, and self‐reported physical and mental health. Mental health impact was assessed by the 8‐item version of Personal Health Questionnaire Depression Scale (PHQ‐8).

This study was conducted in accordance with the Declaration of Helsinki and approved by the Ethics Committee of the Fondazione Policlinico Gemelli of the Università Cattolica del Sacro. All participants gave electronic informed consent for their participation.

Data are available on request from the authors.

### Measures

Our dependent variable is mental health and wellbeing, measured using PHQ‐8 which is established as a valid diagnostic and severity measure for depressive disorders (Kroenke et al., 2009). The PHQ‐8 consists of eight of the nine criteria on which the DSM‐IV diagnosis of depressive disorders is based (American Psychiatric Association, 1994). The ninth question in the DSM‐IV assesses suicidal or self‐injurious thoughts and was omitted because research indicates that the deletion of this question has only a minor effect on scoring, since thoughts of self‐harm are fairly uncommon in the general population (Rief et al., 2004).

The PHQ‐8 asks for the number of days in the past 2 weeks that the respondent had experienced a particular depressive symptom along a range that spans from 0 (never – 0–1 days) to 3 (nearly every day – 12–14 days). The items are: (1) Little interest or pleasure in doing things; (2) Feeling down, depressed, or hopeless; (3) Trouble falling or staying asleep, or sleeping too much; (4) I Feeling tired or having little energy; (5) Poor appetite or overeating; (6) Feeling bad about yourself – or that you are a failure or have let yourself or your family down; (7) Trouble concentrating on things, such as reading the newspaper or watching television; and (8) Moving or speaking so slowly that other people could have noticed? Or the opposite – being so fidgety or restless that you have been moving around a lot more than usual (PHQ‐8 question is reported in Table A1 in Appendix 1).

The scores for each item are summed to produce a total score between 0 and 24 points. A total score of 0 to 4 represents no significant depressive symptoms. A total score of 5 to 9 represents mild depressive symptoms; 10 to 14, moderate; 15 to 19, moderately severe; and 20 to 24, severe (Kroenke et al., 2009). Cronbach's Alpha for the PHQ‐8 was *α* = 0.87 in the current sample. Current depression was defined as a PHQ‐8 score of ≥10, which has an 88% sensitivity and 88% specificity for major depression (Kroenke and Spitzer, 2002) and, regardless of diagnostic status, typically represents clinically significant depression (Kroenke et al., 2009).

The independent variable is quintile of risk perception associated with the pandemic, measured using five variables: (a) scared of getting sick (which range from 1 ‘Not at all’ to 10 ‘Very much); (b) scared that a loved one, family member or friend will fall ill (from 1 ‘Not at all’ to 10 ‘Very much’); (c) the probability of contracting the disease from COVID‐19 (from 1 ‘Not at all probable’ to 5 ‘Very probable’); (d) the degree of control over contracting the infection (1 ‘Not at all’ to 10 ‘Completely’); (e) the fear of not surviving in the event of contracting COVID‐19 (scale from 1 ‘Not at all’ to 10 ‘Completely’). A risk perception index was constructed as the sum of the scores attributed by individuals on the five variables of interest. The range of the scale varies from 5, minimum risk perception, to 45, maximum risk perception. Finally, for each country separately, the risk perception index quintile was computed.

In multivariate models, we used a set of control variables to obtain parameter estimates. First, we included a measure of perceived health. Respondents’ self‐assessed health was initially rated on a 5‐point scale from ‘very good’ to ‘very bad’, and participants were subsequently dichotomized as healthy individuals if they declared a ‘very good,’ ‘good’ or ‘fair’ health status, or unhealthy if they declared a ‘bad’ or ‘very bad’ health status. Second, we use the respondent's reported city‐size for controlling for urban density, coded into six groups (from fewer than 10,000 inhabitants to 500,000 or more inhabitants). Third, we included a temporal predictor – the number of months that had passed since the end of lockdown at the time the questionnaire was completed, with the exception of Sweden which had no lockdown. In this respect, we hypothesized that the longer the time that had elapsed since the end of the lockdown, which was assumed to have been the most severe period of the pandemic, the lower people's risk perception when they filled out the questionnaire. Fourth, we included three variables as predisposing factors: age in four classes (16–29 years; 30–49 years; 50–64 years; 65 or more years), gender and marital status (single; in a relationship or cohabiting; married or civil partnership; separated/divorced or widowed). The fifth type of confounder was enabling factors, which included two variables. Specifically, these were: educational attainment, rated in three categories, ‘lower secondary or below’, ‘upper secondary or post‐secondary’, and ‘tertiary’; the respondents’ occupational status aggregated into five categories (employed; searching for work; homemaker or fulltime parent; students; not able to work, retired).

Finally, to control for country‐level heterogeneity, three macro‐level variables were considered: national health‐care expenditure (all financing schemes) as a percentage of Gross Domestic Product (GDP), which varies by country from 6.3% in Poland to 11.2% in France; the number of hospital beds for acute care, per 1000 inhabitants, which varies from 2.0 in Sweden to 4.8 in Poland; and the number of medical specialists per 100,000 inhabitants, which varies from 76.8 in France to 155.4 in the Czech Republic. These indicators are included in the OECD Framework for the measurement of health system performance (Arah et al., 2006). Thus, we hypothesize that the negative impact of risk perception was less in countries with better indicators of the health system's performance, i.e. these indicators play a moderating effect on the relationship that we are studying.

Descriptive statistics for the dependent variable series and predictor variables are shown in Appendix 1: Table A2.

### Statistical analysis

The obvious strategy for analysing hierarchical data of this type, whereby individuals are nested in countries, would be multilevel regression modelling. Multilevel modelling is associated with two problems with small numbers (*N* < 30) of estimated models of macro‐level units (Bryan & Jenkins, 2016). First, biased estimates of second‐level standard errors are implied by a small sample size at level two (Cora et al., 2005). Second, with limited degrees of freedom at the country level, a restricted number of macro‐indicators can be subject to omitted variable bias (Möhring, 2012).

As an alternative to multilevel methods, we adopted the fixed‐effects approach (Allison, 2009) for country comparisons when the number of second‐level units is limited. Deploying a fixed‐effects estimation means that a country‐specific error term is explicitly estimated, and belongs to the fixed part of the equation.

Formally:

$$y_{\mathrm{ij}}=\gamma_{00}+\beta_{1}x_{1ij}+\cdots +\beta_{k}x_{\mathrm{kij}}+\delta_{1}x_{1\mathrm{ij}}u_{j1}+\cdots +\delta_{N‐1}x_{1\mathrm{ij}}u_{jN‐1}+\alpha_{1}u_{j1}+\cdots +\alpha_{N‐1}u_{jN‐1}+e_{\mathrm{ij}}$$

where *y_ij_* is the individual‐level dependent variable of observation *i* in country j; *γ*_00_ is the intercept over all countries (note that the country‐specific intercept *γ*_0_ *_j_* equals *γ*_00_ + *u_j_*); *x_kij_* is the independent individual‐level variable number *k*; *β_k_* is the coefficient on the individual‐level variable number *k*; *u_j_* is the error term for each country *j*; and *e_ij_* is the error term for observation *i* within country *j*.

In this analysis four models were estimated.

Model 1 (M1) was devised to evaluate how much variance can be explained by the second level. M1 includes only *N*−1 dummy variables for the individual countries. The coefficient of determination (R2) tells us the percentage of variance that is due to country‐level variation. Model 2 (M2) added the independent variable (risk perception) and micro‐level predictors (individual variables). Model 3 (M3) tested whether the effects of risk vary across countries (equivalent to the ‘slope effect’ in multilevel models). Interaction terms for risk and country dummies were added to M3. The final step was Model 4 (M4) that added the cross‐level interaction effect (that is, interactions between micro and macro variables). The use of macro‐cross‐level interaction terms allows a moderator effect of macro variables on individual characteristics to be estimated.

## RESULTS

Our sample was not recruited through probabilistic criteria (see Limitation paragraph) which has to be taken into account in interpreting these findings.

**FIGURE 1:**
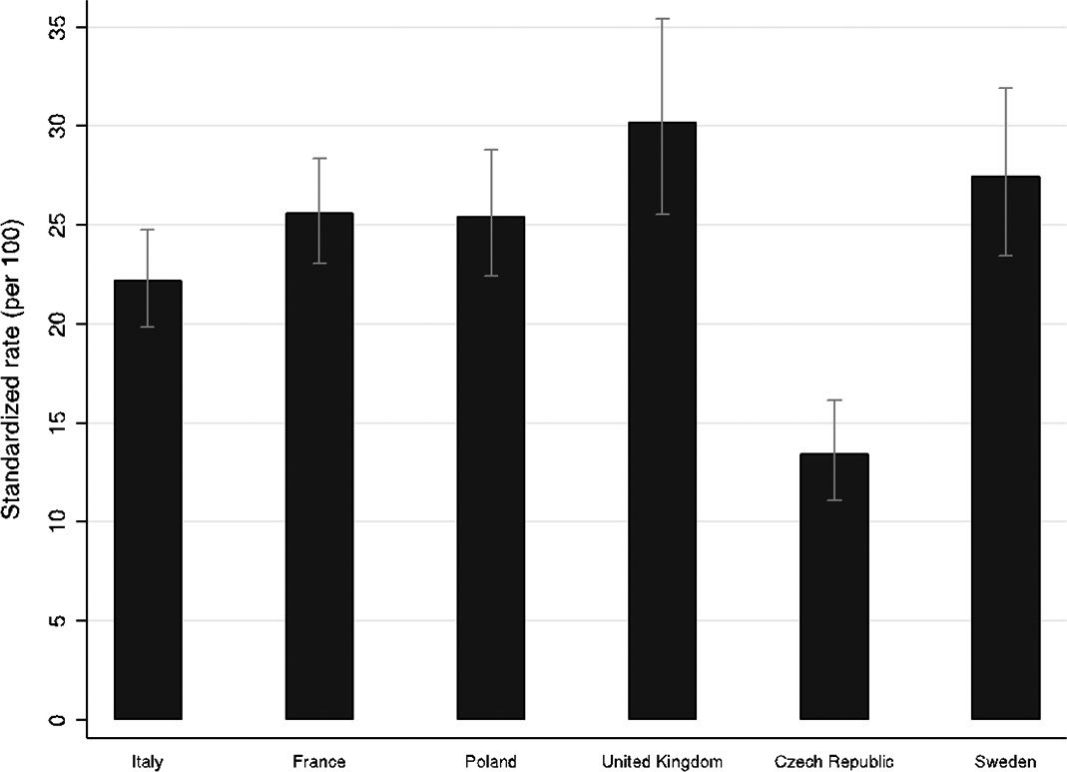
Age standardized prevalence of depressive symptoms (PHQ‐8 ≥ 10 points) during the last two weeks by country. PHQ‐8 Index variable was dichotomized: depression =≥10 points; age was codified here as a continuous variable between 21 and 80 years

The mean age of the participants was 42.43.09 ± 15.05 years. The majority of respondents were women (70.2%), married (43.0%), with a household size of 3–5 people (80.7%), with children (60.0%), employed (64.7%), and well educated (52.5% ≥ bachelor's degree). In Figure 1, we report the standardized rate of the PHQ‐8 Index as being more than 10, which is indicative of a depressive symptomatology (Kroenke et al., 2009), adjusted for age by country. We found significant differences across countries.

The United Kingdom had the worst level of wellbeing (i.e. an adjusted rate of 30.2, confidence interval (CI) = 25.6–35.4), followed by Sweden (27.5; CI 23.5–31.9) and France (25.6; CI 23.1–28.4) and Poland (25.5; CI 22.4–28.8). In contrast, the proportion of people in the Czech Republic with a PHQ‐8 Index greater than 10 is 13.5 (CI 11.1–16.2). These results show a significant difference in depression compared with the pre‐pandemic period. According to Eurostat data (2020), in 2017 the share of population reporting that they had chronic depression was 9.6% in Sweden, 8.9% in United Kingdom, 5.9% in France, 5.5% in Italy, 4.2% in Poland, and 3.9% in the Czech Republic.

**FIGURE 2:**
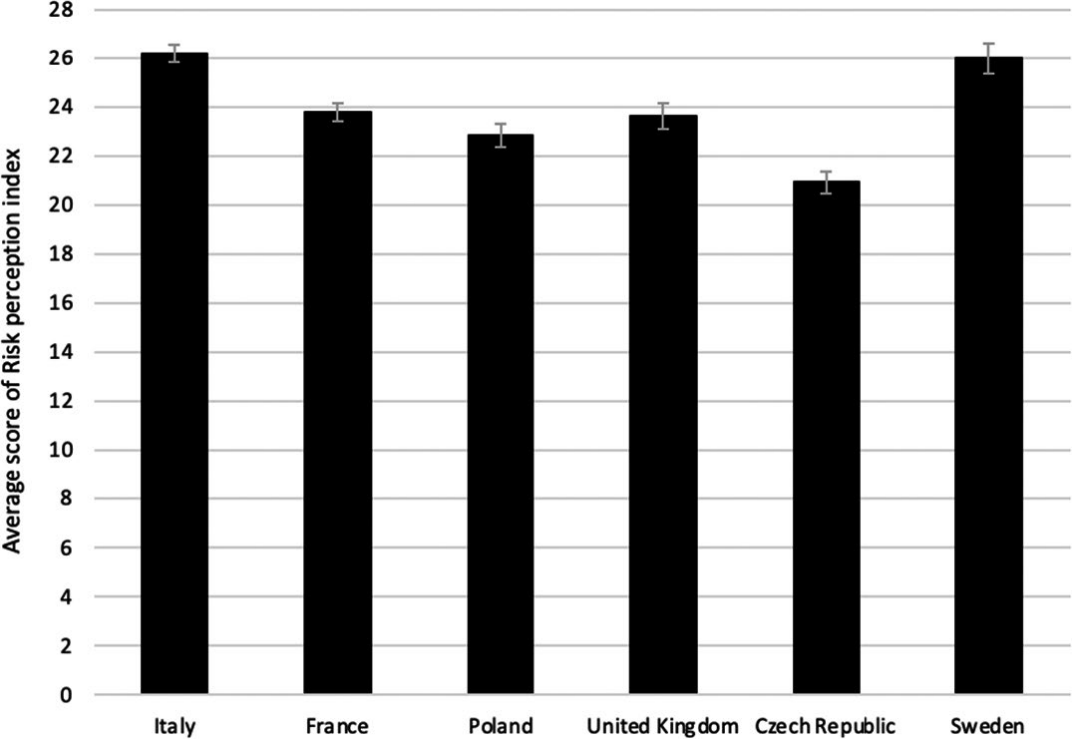
Average score of risk perception index and 95% confidence intervals by country. Risk perception index varies between 5 and 45 points

Moreover, significant differences in risk perception among countries were found (Figure 2): the Czech Republic (20.9, CI 20.5–11.4) showed the lowest level of risk perception, whereas Italy (26.2, CI 25.8–26.6) and Sweden (26.0, CI 25.4–26.6) displayed the highest level of risk perception, with other countries in between.

Using this framework, we estimated whether, and to what extent, subjective risk perception affects people's mental wellbeing. The estimated multivariate M1 (data not shown) includes *N*−1 dummy variables to represent individual countries. The R2 value, representing the variance explained by country level, was very low: 2.6%. M2 also includes individual‐level variables and explained considerably more of the variance: 25.0%. To evaluate whether the introduction of micro‐level variables in M2 significantly improved the fit of the model compared with M1, a likelihood‐ratio test (LR test) was used. The likelihood‐ration test indicated that M2 improved the prediction relative to M1 (see Table 1).

**TABLE 1:** Likelihood‐ratio test to compare models

	M2‐M1	M3‐M2	M4_EXP‐M2	M4_BED‐M3	M4_MED‐M2
Likelihood‐ratio test					
Difference	7255.48	122.32	8.67	11.27	8.45
Degree of freedom	25	20	4	4	4
Sig.	0.00	0.00	0.07	0.02	0.08

The estimated effect of the variable of interest—specifically, our measure of risk perception—is shown in Table 2: the higher the degree of risk perception, the worse the level of mental health. Even after controlling for confounders, individuals coded in the last quintile of the risk perception index displayed a level of depression that was about three points higher than the level of depression of those who were in the first quintile. Risk perception, as we have defined it, therefore, has a significant association with self‐reported mental health.

**TABLE 2:** Fixed‐effects estimation of Model 2. The association between risk perception and PHQ‐8 Index in six European countries. Beta parameters, 95% interval confidence and significance levels (*p*‐value)

Model 2	Beta	CI
Quintile of risk perception index		
First quintile risk perception	Ref.	
Second quintile risk perception	0.62***	0.30 to 0.94
Third quintile risk perception	1.32***	1.00 to 1.64
Fourth quintile risk perception	1.90***	1.58 to 2.22
Fifth quintile risk perception	3.10***	2.77 to 3.42
Country		
Italy	Ref.	
France	0.65**	0.26 to 1.05
Poland	0.19	−0.34 to 0.72
United Kingdom	1.64***	0.78 to 2.51
Czech Republic	−1.82***	−2.20 to −1.44
Sweden	0.70	−0.23 to 1.63
Gender		
Male	Ref.	
Female	0.77***	0.56 to 0.97
Age in class		
16–29 years	Ref.	
30–49 years	−1.60***	−1.96 to −1.25
50–64 years	−3.10***	−3.49 to −2.71
65+	−4.61***	−5.12 to −4.10
Level of education		
Low	Ref.	
Medium	−0.03	−0.29 to 0.24
High	−0.36*	−0.68 to −0.05
Occupational condition		
Employee	Ref.	
Looking for job	0.44*	0.01 to 0.88
At home	0.99***	0.45 to 1.53
Student	1.38***	0.94 to 1.81
Retired	1.02***	0.68 to 1.36
Marital status		
Single, never married	Ref.	
In a relationship and cohabitant	−1.15***	−1.46 to −0.83
Married or registered partnership	−1.03***	−1.37 to −0.70
Separated/divorced or widowed	−0.20	−0.63 to 0.23
City‐size		
500,000 +	Ref.	
250,000–499,999	−0.89***	−1.37 to −0.41
100,000–249,999	−1.02***	−1.39 to −0.66
20,000–99,999	−0.81***	−1.15 to −0.48
10,000–19,999	−1.00***	−1.37 to −0.62
Less than 10,000	−1.28***	−1.59 to −0.97
Have children		
Yes	Ref.	
No	−0.22	−0.48 to 0.05
Perceived health status		
Good	Ref.	
No good	2.98***	2.75 to 3.21
Interview time		
Months from lockdown	0.01	−0.00 to 0.02
Constant	6.65***	5.91 to 7.38
Observations	8495	
Log‐likelihood	−24950.844	
*R* ^2^	0.25	

Note

Significance levels: ***p < 0.001, **p < 0.01, *p < 0.05.

Looking at other variables in the model, we observe that individuals with poor levels of self‐assessed health scored about three points more than those in good health. Also, the city dimension counts: people who lived in small towns have a lower level of depression than individuals who stay in big cities. With regard to predisposing factors, we observe that age was associated with the PHQ‐8 index: older people show better mental health conditions than younger individuals. Having children is not a statistically significant parameter, whereas being married or in a relationship reduces the level of depression. Moreover, women had lower levels of wellbeing, by around 0.8 points, compared with men. A set of variables related to enabling factors were included in the model and both education and occupation were associated with mental wellbeing. Compared with the employed, other occupational statuses show a higher level of depression (around one point). By contrast, people with a higher education level have 0.4 point less in the depression index than people with lower education.

Rather than assuming, as we did in M2, that the impact of risk perception was the same for each country, we tested whether the effect of risk perception varies across countries with a new model, M3 (data not shown), which included interaction effects of the country dummies and the measure of individual risk perception. These interaction effects (equivalent to the so‐called ‘slope effect’ in multilevel models) permitted assessment of differences in the relationship between risk perception and depression across countries. Comparing M3 with M2 with the Likelihood Ratio (LR) test, we find (see Table 1) that M3 (namely, the model with interaction effects) should be preferred over M2 (that is, the model that does not include interaction effects between the risk perception and country dummy variables). From a substantive point of view, this means that the association between risk perception and depression could be different in the various countries included in this study. Higher risk perception is associated with lower mental wellbeing, but the form and strength of this relationship varies between countries.

**FIGURE 3:**
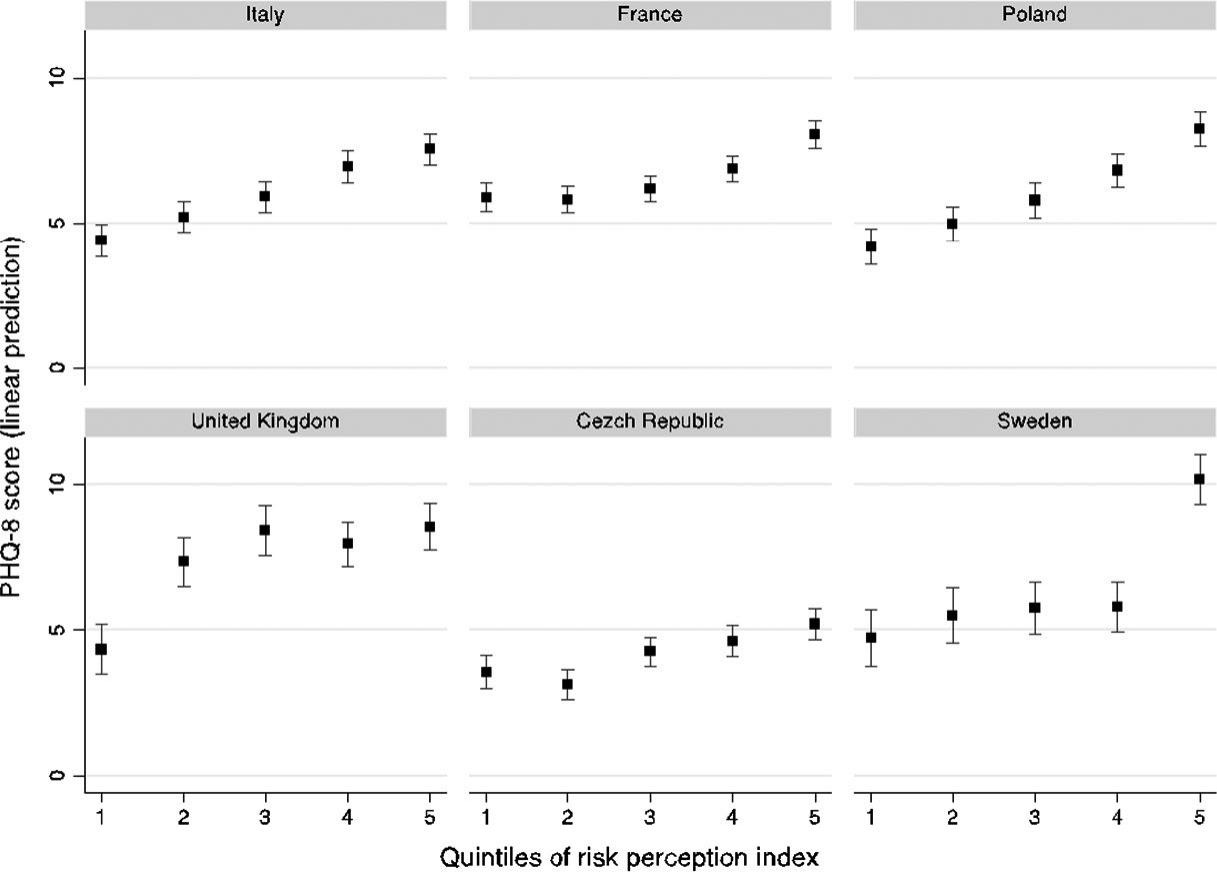
Fixed‐effect Model 3, depression in European countries. Average marginal effects and 95% confidence interval of interaction between quintile risk perception and country

To display this result, Figure 3 illustrates the predictive margins of risk perception and country interaction as indicated by M3. As we can see, the effect of risk perception differs considerably between countries. In general, we noted that individuals with higher perception of risk had a lower score on the PHQ‐8 index than those with a lower degree of risk perception. However, while Italy, Poland, and partially in France, a clear gradient was observed, i.e. any increase in quintile of perception of risk was associated with a worsening in the depression index, in the United Kingdom, the Czech Republic, and Sweden, this relationship is very different. In the UK, people from the second to the fifth quintile of risk perception showed the same high level of depression (differences are not statistically significant) and only those in the first quintile had a lower degree of depression; in the Czech Republic, differences between people with dissimilar levels of risk perception are minimal, only the first two quintiles seem to show better mental health; finally, in Sweden only people with the highest degree of risk perception exhibit a relevant score in PHQ‐8 depression index compared with individuals in the other fourth quintiles, which have all the same level. However, interaction effects contributed very slightly to explaining the differences in the level of mental wellbeing. The increase of explained variance in passing from M2 to M3 was very modest ‐ 25.0 to 26.1.0%.

The disparities that we observe between countries could be due to social, economic, and/or public policy differences, that is the context that varies across countries. To address this, we evaluated the moderator effect of context (i.e. health‐care expenditure as a percentage of Gross Domestic Product (GDP), hospital beds for acute care and number of medical specialists per head of population) on the relationship between risk perception and depressive symptoms. These different institutional arrangements could reduce the effect of risk perception on depression if people evaluated that health care could help them when they need it, specifically if they got sick. To investigate this question, three further models were developed: M4_EXP includes the interaction between risk perception and the health‐care expenditure; M4_BED includes the interaction between risk perception and acute hospital beds; finally, M4_MED includes the interaction between risk perception and the number of medical specialists. Also, in this case, we initially compared models by Likelihood Ratio (LR) test of M4_ EXP, M4_ BED and M4_ MED with those from M2.

The differences detected in the LR test show that the goodness‐of‐fit of the M4_BED models was higher than that of M2 (see Table 2); therefore, we can state that the availability of hospital beds for acute care moderates the relationship between risk perception and depression: the higher the number of acute hospital beds (per 1000 inhabitants), the lower the negative impact of perception of risk on mental wellbeing. By contrast, models M4_EXP and M4_MED do not show statistically significant improvement at 0.05 level compared with M2, although the LR results are statistically significant at the 0.1 level. There was evidence that different indicators considered in this study, in particular acute hospital beds, were able to mitigate to different extents of the effect of risk perception on mental wellbeing.

**FIGURE 4:**
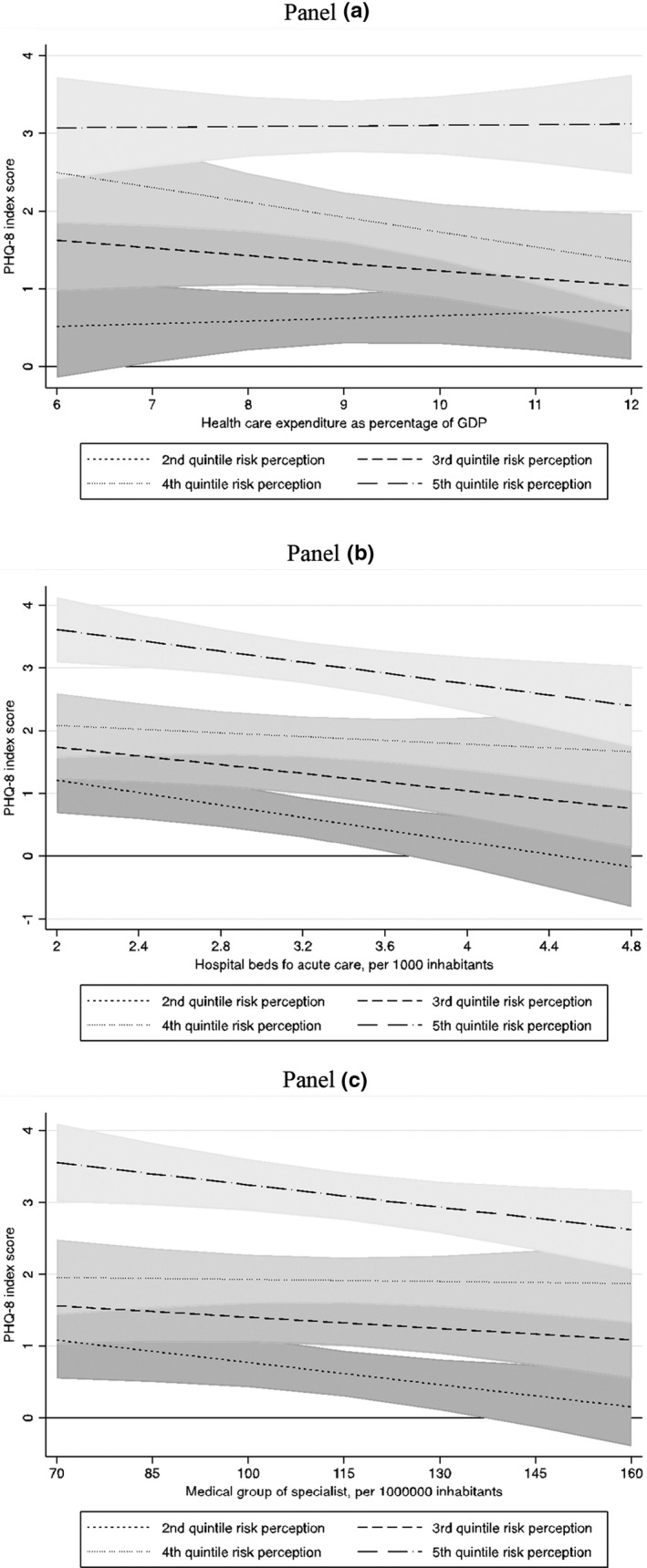
Fixed‐effects estimation of Model 4_EXP (Panel a), Model 4_BED (Panel b), and Model 4_MED (Panel c) mental wellbeing in six European countries. Predictive margins and 95% confidence intervals of interaction between risk perception and health‐care expenditure as percentage of GDP (Panel a), hospital beds for acute care for 1000 inhabitants (Panel b), and number of medical specialists for 100,000 inhabitants (Panel c)

However, looking at Figure 4, which displays the predictive margins of interaction between risk perception and health expenditure (Panel a), between risk perception and the number of acute hospital beds (Panel b), and between risk perception and the number of medical specialists (Panel c), we see some dissimilarity, suggestive of a different capacity of these indicators to reduce the negative impact of risk perception on individuals’ depression.

In particular, the magnitude of inequalities seems to be lower for countries with higher health‐care expenditure, especially for people in the middle quintile risk perception groups (the third and, above all, fourth quintile), whereas the level of health‐care expenditure does not modify the relationship for people with the highest level of perception of risk. With respect to acute hospital beds, we observe that countries with more hospital beds per 1000 inhabitants showed a clear reduction in the depression score, particularly for people in the first and fifth quintile of risk perception, and who lived in countries with lower or medium availability of acute hospital beds. Finally, regarding the number of medical specialists, we can state that the form of the relationship is very similar to that described for hospital beds. Also, in this case, the higher the number of medical specialists, the lower the relationship between risk perception and people's mental wellbeing, in particular for the first and fifth quintile of the risk perception index.

Before discussing the significance of our analysis in light of the specific sampling approach, we summarize the main findings. First, in all countries included in the analysis we observe a high proportion of people suffering from depression (PHQ‐8 score ≥10), from 13% in the Czech Republic to 30% in the UK (age‐adjusted). Second, the perception of risk related to the pandemic varies between countries, i.e. Italy and Sweden show the highest level of perception of risk and the Czech Republic the lowest level. Third, according to the fixed‐effect regression model used, risk perception is associated with depression; the higher the level of risk perception, the greater the rate of depression, controlling for a set of confounding variables. Moreover, this relationship varies significantly between countries: in Italy, Poland, and France a clear gradient is found, whereas in the UK and Sweden differences are seen only for the lowest and the highest level of risk perception index, respectively. Fourth, the association is moderated by contextual factors. Institutional arrangements considered in this analysis (i.e. health‐care expenditure as a percentage of GDP, hospital beds for acute care and number of medical specialists per head of population) reduce, although in different ways, the effect of risk perception on depression.

### Sensitivity analysis

To examine how the baseline results change when different specification and estimation scenarios are applied, we conducted two types of robustness check.

The first concerned our independent variable, perception of risk index. Factor analysis was applied for each country to test whether the variables used for the construction of the index were associated with a single latent construct of risk perception (principal‐component factor with varimax orthogonal rotation of factors undertaken, data not shown). A single latent dimension emerges in all countries, explaining between 48 and 56% of the total variance. Starting with predicted factor score values, we obtained a new index of risk perception, whose correlation with the risk perception additive index used in the baseline model is 0.95, supporting the reliability of the additive index used.

The second type of sensitivity control related to the value of the PHQ‐8 index threshold as an indicator of depression. Studies report a PHQ‐8 cut‐off score of ≥10 as indicating a clinically relevant condition (Kroenke et al., 2009), so we evaluated the sensitivity of the analysis using a dichotomous variable for PHQ‐8 rather than using a continuous variable. The results (data not shown) were very similar to the baseline specification in Table 1 when a continuous dependent variable was used. Considering the first quintile of risk perception index as the reference category, only the odds ratio of the second quintile was not statistically significant, i.e. 1.17 (CI 0.93–1.47), whereas the odds ratio of the third quintile was 1.52 (CI 1.22–1.89), the odds ratio of the fourth quintile was 2.24 (CI 1.81–2.77) and the odds ratio of the fifth quintile of risk perception was 3.40 (CI 2.75–4.20). These findings confirm the association between perceived risk and individuals’ mental health: for example, people with the highest level of perception of risk have greater probability (more than 3 times according to our results) of being depressed than people with the lowest level of risk perception.

## DISCUSSION

Drawing on harmonized data from six European countries, our web‐survey data of the general adult population show depression to be a feature of the experience of the pandemic and of the associated public health measures. Levels of depression self‐reported in our opportunistically sampled survey are higher compared to pre‐pandemic population levels as shown by Eurostat data. The extent to which levels of depression were elevated varied across the six countries, which had differing experience of the pandemic in terms of timing and public health precautions adopted. Furthermore, the level of subjectively assessed perceptions of risk represented by the pandemic also varied significantly across the six countries.

In exploring the relationship between the perception of risk and depression across the six countries sampled, we adopted a fixed‐effects approach to examine the role of individual‐level variables in the cross‐country variance, while controlling for the heterogeneity represented across the six countries.

Our working hypothesis was that where perceptions of risk of the pandemic were elevated, people would be more likely to report depressive symptoms. The extent to which people felt at risk from the pandemic was likely to be a product of a range of different variables that operate both individually and contextually. The variance in the relationship between perception of risk and depression is difficult to account for, given the plethora of variables at individual and aggregate level across different areas of social, political, and cultural life.

At individual level, people with poor levels of general health were far more likely to report poor mental health, a finding which confirms existing understandings of the links between physical and mental health. Other factors that predispose to poor mental health that were confirmed in this study were younger age, being single and living in a bigger city (in contrast to those in smaller cities or towns). Higher levels of education and being employed seemed to be protective of mental health.

Moving to the aggregate level, our findings show that Italy and Sweden demonstrated the highest level of perception of risk, and the UK and Sweden had the worst levels of mental health reported. Nonetheless, the ways that perception of risk influenced mental health varied across the six countries. Across the whole sample, having a higher perception of risk was positively associated with reported depression and this relationship was true across all six countries, although the dimensions and strength of the association varied. So, while the association between lower perception of risk and better mental health was a clear gradient in Italy, Poland, and France to some extent, the relationship in Sweden, the Czech Republic, and the UK could not be characterized so simply.

In accordance with our theoretical framework, risk perception depends not only on the pandemic and public health measures, but also on socio‐cultural context, one element of which is trust (Maturo, 2004). Trust can be seen as a social fact, an attribute of social conglomerates – communities and nation‐states (Hadis, 2014), that affects many social discourses, including the level of confidence in organizations such as hospitals (Sztompka, 2000).

During the early months of the pandemic infection and mortality rates, as well as public health measures, were widely reported and widely discussed through official and informal channels. We therefore hypothesized that shared discussion of the performance of the health system influenced people's perception of risk. According to IPSOS survey ‘Global Views On Healthcare’ (2018), trust in health care varies across countries, with the percentage of individuals who state they trust the health system to provide them the best treatments was 36% in Italy, 50% in France, and 63% in the UK. We examined the moderating effect of the national level of health‐care expenditure and availability of hospital care on the positive association between risk perception and depression. This modelling suggested that a higher availability of hospital beds per head of population reduces the positive effect of risk perception on depression levels. The mitigating effects of higher overall health‐care expenditure and the higher availability of medical specialists were not so strong, but followed the same pattern as that for acute bed availability.

The mitigating effects of the higher availability of hospital beds on elevated risk perception's positive association with depression seem to work best for countries with higher total levels of health‐care expenditure and especially for those people reporting the middle quintiles of risk perception. That is to say, those with the highest perceptions of risk were least affected by these contextual factors. For people with highest and lowest levels of risk perception, the depression score was reduced in countries with the highest number of acute hospital beds per head of population. There was evidence of a similar relationship for the number of medical specialists per head of population since in countries with the highest numbers of specialists the effect of perception of risk on depression was reduced.

## CONCLUSION

These analyses have begun to untangle the relationship between individual‐level perception of risk, depression, other individual‐level variables, and the effects of aggregate‐level contextual factors. Our findings confirm that contextual factors related to both infrastructure and to trust in government policy have an effect on individual mental health symptoms, even among young and healthy populations (Tasso et al., 2021). We offer evidence that generously funded health‐care systems can moderate the effect of risk perceptions on elevated rates of depression during pandemic restrictions. Understanding the effects of contextual factors is crucial, not only for addressing and counteracting depression in the medium and long term, but also to counter an excessive focus on the individual as the locus of intervention through concepts such as resilience (Marshall et al., 2020; Prime et al., 2020).

While these are tentative findings, based on a web‐based survey that was undertaken during a very difficult period of disruption engendered by the first wave of the COVID‐19 pandemic, we believe that they offer important clues towards refining pandemic public health measures. The early months of the pandemic were characterized by intense research activity around the nature of the virus and policy efforts were directed towards measuring infection, morbidity and mortality rates, as well as establishing public health precautions and clinical care protocols. Efforts to communicate the risk of transmission through orthodox and novel channels, aimed at promoting compliance with preventive precautions, may also have exacerbated depressive symptoms among those who are particularly susceptible (Wheaton et al., 2021). These unintended negative effects of public health infection‐prevention policies, putting up depressive morbidity rates, have been far less intensively studied, despite the high mental health costs of lockdown being widely acknowledged by health‐care and welfare providers. One line of future research is how social groups are differentially affected by these effects. Our results underline the importance of public health planning that goes beyond infection and mortality rates to assess public health measures in context. Investment in health‐care infrastructure, in the form of hospital beds and medical specialists, seems to reduce the damaging effect of risk perception on mental health, giving a clear concrete means of protecting the population from depressive symptoms in pandemic times. Clearly, further research will be required to confirm our findings.

## LIMITATION

Several methodological limitations should be considered when interpreting the results of this study. Relying on voluntary recruitment and snowball sampling via social media could have introduced important selection bias: first, by excluding people not on social media; second through self‐selection bias, as suggested by the highly unbalanced gender and education ratios observed; and third, by allowing multiple responses to the survey, although this issue has mainly been documented for surveys with incentives (Cobanoglu & Cobanoglu, 2003), and this was not the case in our survey. The oversampling of respondents with high levels of education and Internet access may have underestimated the burden of poor mental health, particularly in countries with poorer internet access infrastructure. In order to control these biases and reduce the coverage error, we used post‐stratification techniques (Schonlau & Couper, 2017).

Furthermore, the problem of reversed causality should be considered. For example, depressed individuals choose to isolate themselves, and lack of support could increase perception of risk; moreover, depressed individuals will tend to give more extreme and negative responses to survey questions. One potential (albeit imperfect) solution to the causality problem is to separate the measurement in time by looking at people with high‐risk perception and non‐depressed individuals at *t* and studying their probability of becoming depressed at *t* + *1* relative to those with low perception risk at *t*. This approach to establishing causality was not possible to pursue in our analysis which was designed to uncover correlations between variables.

## CONFLICT OF INTEREST

The authors declare that they have no conflict of interest.

## AUTHORS’ CONTRIBUTIONS

**Marco Terraneo:** Conceptualization (equal); data curation (supporting); formal analysis (lead); investigation (supporting); methodology (equal); project administration (supporting); writing – original draft (equal); writing – review and editing (supporting). **Lombi Linda:** Conceptualization (equal); data curation (lead); formal analysis (supporting); investigation (lead); methodology (equal); project administration (lead); writing – original draft (equal); writing – review and editing (supporting). **Hannah Bradby:** Conceptualization (equal); investigation (supporting); methodology (supporting); writing – original draft (equal); writing – review and editing (lead).

## ETHICAL APPROVAL

The study was approved by the Ethics Committee of the Policlinico Universitario A. Gemelli IRCCS of Università Cattolica del Sacro Cuore (prot. 0025523/20).

## Data Availability

The datasets are available from the corresponding author on request.

## APPENDIX 1

**TABLE A1:** The PHE‐8 Scale

How often during the past 2 weeks were you bothered by…				
	Never (0–1 days)	Several days (2–6 days)	More than half the days (7–11 days)	Nearly every day (12–14 days)
(a) … Little interest or pleasure in doing things?	●	●	●	●
(b) … Feeling down, depressed, or hopeless?	●	●	●	●
(c) … Trouble falling or staying asleep, or sleeping too much?	●	●	●	●
(d) … Feeling tired or having little energy?	●	●	●	●
(e) … Poor appetite or overeating?	●	●	●	●
(f) … Feeling bad about yourself, or that you are a failure, or have let yourself or your family down?	●	●	●	●
(g) … Trouble concentrating on things, such as reading the newspaper or watching television?	●	●	●	●
(h) … Moving or speaking so slowly that other people could have noticed. Or the opposite – being so fidgety or restless that you have been moving around a lot more than usual?	●	●	●	●

**TABLE A2:** Descriptive statistics of variables employed in regression models

	PHQ‐8 Score	Quintile of risk perception index	Gender	Age in class	Level of education	Occupational condition	Marital status	City‐size	Have children	Perceived health status	Interview time
Italy											
Mean	6.0	3.1	1.5	2.4	1.7	2.3	2.4	4.3	1.4	0.3	39.8
*SD*	4.6	1.4	0.5	0.9	0.7	1.6	1.0	1.6	0.5	0.4	3.1
Min	0	1	1	1	1	1	1	1	1	0	26
Max	24	5	2	4	3	5	4	6	2	1	44
France											
Mean	6.7	3.1	1.5	2.3	2.1	2.1	2.4	4.5	1.4	0.3	13.5
*SD*	5.5	1.4	0.5	0.9	0.7	1.6	1.0	1.6	0.5	0.5	10.2
Min	0	1	1	1	1	1	1	1	1	0	−44
Max	24	5	2	4	3	5	4	6	2	1	27
Poland											
Mean	5.8	3.0	1.4	2.5	1.8	2.4	2.5	3.6	1.4	0.3	−1.2
*SD*	5.6	1.4	0.5	1.0	0.4	1.8	1.0	1.7	0.5	0.5	18.6
Min	0	1	1	1	1	1	1	1	1	0	−49
Max	24	5	2	4	2	5	4	6	2	1	25
UK											
Mean	7.2	3.1	1.5	2.2	2.2	1.9	2.5	3.7	1.4	0.2	−38.1
*SD*	6.0	1.4	0.5	0.9	0.7	1.5	0.9	1.8	0.5	0.4	2.9
Min	0	1	1	1	1	1	1	1	1	0	−58
Max	24	5	2	4	3	5	4	6	2	1	−26
Czech Republic											
Mean	4.4	3.1	1.5	2.4	2.1	2.7	2.5	3.1	1.4	0.3	20.1
*SD*	4.6	1.4	0.5	1.1	0.6	1.9	1.0	2.2	0.5	0.5	3.8
Min	0	1	1	1	1	1	1	1	1	0	5
Max	24	5	2	4	3	5	4	6	2	1	25
Sweden											
Mean	6.5	3.1	1.5	2.4	2.3	2.1	2.3	3.6	1.6	0.4	−44.0
*SD*	6.0	1.4	0.5	0.9	0.7	1.7	1.0	1.8	0.5	0.5	16.1
Min	0	1	1	1	1	1	1	1	1	0	−61
Max	24	5	2	4	3	5	4	6	2	1	−8
